# Conformational Analysis and Structure-Altering Mutations of the HIV-1 Frameshifting Element

**DOI:** 10.3390/ijms26136297

**Published:** 2025-06-30

**Authors:** Katelyn Newton, Shuting Yan, Tamar Schlick

**Affiliations:** 1 University of Portland, Portland, OR 97203, USA; newtonk25@up.edu; 2 Department of Chemistry, New York University, 100 Washington Square East, Silver Building, New York, NY 10003, USA; aimeesty@gmail.com; 3 Courant Institute of Mathematical Sciences, New York University, 251 Mercer St., New York, NY 10012, USA; 4 New York University-East China Normal University Center for Computational Chemistry, New York University Shanghai, Shanghai 200122, China; 5 Simons Center for Computational Physical Chemistry, New York University, 24 Waverly Place, Silver Building, New York, NY 10003, USA

**Keywords:** frameshifting element, viral frameshifting, HIV, RNA-As-Graphs, ribosomal translation, RNA folding, RNA mutations, conformational landscape

## Abstract

Human immunodeficiency virus (HIV) continues to be a threat to public health. An emerging technique with promise in the context of fighting HIV type 1 (HIV-1) focuses on targeting ribosomal frameshifting. A crucial –1 programmed ribosomal frameshift (PRF) has been observed in several pathogenic viruses, including HIV-1. Altered folds of the HIV-1 RNA frameshift element (FSE) have been shown to alter frameshifting efficiency. Here, we use RNA-As-Graphs (RAG), a graph-theory based framework for representing and analyzing RNA secondary structures, to perform conformational analysis in motif space to propose how sequence length may influence folding patterns. This combined analysis, along with all-atom modeling and experimental testing of our designed mutants, has already proven valuable for the SARS-CoV-2 FSE. As a first step to launching the same computational/experimental approach for HIV-1, we compare prior experiments and perform SHAPE-guided 2D-fold predictions for the HIV-1 FSE embedded in increasing sequence contexts and predict structure-altering mutations. We find a highly stable upper stem and highly flexible lower stem for the core FSE, with a three-way junction connecting to other motifs at increasing lengths. In particular, we find little support for a pseudoknot or triplex interaction in the core FSE, although pseudoknots can form separately as a connective motif at longer sequences. We also identify sensitive residues in the upper stem and central loop that, when minimally mutated, alter the core stem loop folding. These insights into the FSE fold and structure-altering mutations can be further pursued by all-atom simulations and experimental testing to advance the mechanistic understanding and therapeutic strategies for HIV-1.

## 1. Introduction

In 2022, approximately 39.0 million individuals were living with human immunodeficiency virus (HIV) worldwide [1]. The infection attacks the body’s white blood cells, leading to reduced immune response and putting individuals at risk for developing acquired immunodeficiency syndrome (AIDS), the most severe stage of infection. While treatment is available, a cure or long-term solution has yet to be found. With over 600,000 deaths related to AIDS in 2022, nearly two-thirds of them occurred in sub-Saharan Africa [1]. Such a public health crisis requires new antiviral strategies. For example, SARS-CoV-2 related work by the RNA-Puzzles Consortium focused on the modeling and comparative analysis of conserved viral RNA regions [2]. Another area of recent interest relating to the SARS-CoV-2 virus and other viral infections is the disruption of highly conserved regions in the RNA genome. See, for example, recent reviews on frameshifting elements and their usage as potential therapeutic targets [3,4,5,6], as well as [7,8,9,10,11,12,13,14,15,16] for HIV-1 and SARS-CoV-2. The current treatment of HIV-1 is dominated by combined antiretroviral therapy (cART); an estimated 60% of HIV-1 patients across the globe received a form of this treatment in 2018 [17]. Such cocktails target replication of the virus, but antiretroviral therapy is vulnerable to drug resistance, adaptations, financial, and life-long burdens [18]. Identifying target residues for gene editing or small molecule inhibitors within the frameshift element (FSE) could potentially attack the source of the virus itself and provide long-term treatment options. In viral RNA, programmed ribosomal frameshifting (PRF) facilitates alternative viral protein products by utilizing shifted reading frames, a strategy that can enhance product variety for compact viral genomes [19]. The site of this shifted translation event defines a region for identifying such pivotal residues.

Many crucial HIV-1 enzymes including protease, reverse transcriptase, and integrase are synthesized following the cleavage of the Gag-Pol precursor [20]. Between 90% and 95% of the time, the Gag gene is encoded, but in the remaining translation events, a –1PRF, causes the ribosome to encounter the stop codon for Gag, leading to the synthesis of Gag-Pol polyproteins [20]. The frameshift efficiency in HIV-1 is 5–10%, and such rate of frameshifting is essential to the survival of the virus and, thus, strictly regulated. A study on the maintenance of the Gag/Gag-Pol ratio found that increased local stability in first few base pairs of the FSE results in a significant increase in the frameshift efficiency [21]. This increased stability of the first few base pairs of the FSE caused an increase in frameshift efficiency, which has been correlated with decreased relative infectivity. Conversely, there were no significant changes in the frameshift efficiency with a decrease in the local stability of the FSE [21]. Other studies have suggested frameshifting to be a key target of antiviral strategies for HIV and SARS viruses [8,10,22].

The HIV-1 genome contains between 9200 and 9600 nucleotides [23]. Two key structural elements must be in place for frameshifting to occur, though the actual mechanism has not yet been elucidated: the slippery sequence (U UUU UUA) [24] and stimulatory sequence, or frameshifting element (FSE). Viral frameshifting often depends on a specific secondary structure of the FSE, commonly, a stem loop or pseudoknot. This fold can slow translation and, thereby, induce a shift in the reading frame [25]. Such pausing in frameshifting can accommodate a variety of other secondary structures for one virus FSE [26], as we showed recently for SARS-CoV-2 [27,28,29]. Early models of the HIV-1 FSE proposed structures containing a pseudoknot and a triplex helix [30,31], but other two-stem structures based on NMR data have contradicted those findings [32,33]. A comprehensive analysis of the HIV-1 genome from selective 2′-hydroxyl acylation analyzed by primer extension (SHAPE) data suggests that HIV-1 contains a three-helix junction in which one of the helices is formed by the slippery sequence [34]. Because the structure of the frameshift stimulatory sequence is predicted to have a large impact on frameshift regulation [35], predictive mutations that disrupt those FSE folds are of great interest, and a mechanistic understanding of frameshifting, including the roles of alternate folds, is important.

Here, we augment current knowledge of the HIV-1 FSE with a conformational and mutational analysis of the HIV-1 FSE using the RNA-as-graphs (RAG) representation to the RNA 2D structure. The RAG dual graphs have been used to model, predict, and create RNA secondary structures by simplifying the description of the secondary structure as a combination of vertices and edges [36,37]; note that 2D folding is still performed in nucleotide space. The motifs allow us to group similar folds together, and atomic-level modeling can then be performed to complete the picture [38,39]. Such combined analysis has already shown to be successful for the SARS-CoV-2 FSE: RAG analysis [10,29] with SHAPE and DMS analysis [27,29] and all-atom molecular dynamics, enhanced sampling, and phylogenetics [38,39,40] have confirmed our structure-altering mutants and their one-order-of-magnitude reduction on frameshifting [28].

**Figure 1:**
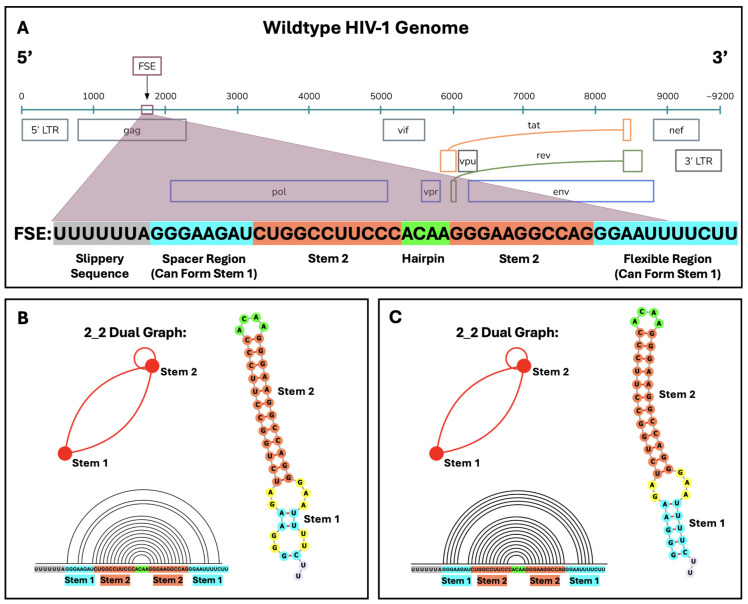
The location of the frameshift element (FSE) within the wildtype HIV-1 RNA and the different reading frames of the HIV-1 genome. The slippery sequence and 45-nucleotide FSE are expanded below the reading frames. (**A**) The stable upper stem (Stem 2) and hairpin, and a spacer region that can base pair with downstream residues to form a flexible lower stem (Stem 1) define the FSE. Despite the fluidity of Stem 1, the core FSE adopts a 2_2 dual graph motif in most cases (see Methods on notation and https://www.biomath.nyu.edu/dual_vertices.php?v=2 for motif library), shown in two base-pairing forms, as obtained from our SHAPE-free (**B**) and SHAPE-guided (**C**) landscapes of secondary structure. Despite differences in the size of the lower stem, both predictions correspond to a 2_2 dual graph, indicating key elements of the general folding pattern. Arc plots of SHAPE-free (**B**) and SHAPE-guided (**C**) folds are produced by R-chie [47] and offer another visualization of the 2_2 core FSE. The hairpin loop is in green, Stem 1 is blue, Stem 2 is coral, internal loops and bulges are yellow, and additional free nucleotides are lavender in 2D structures.

**Figure 2:**
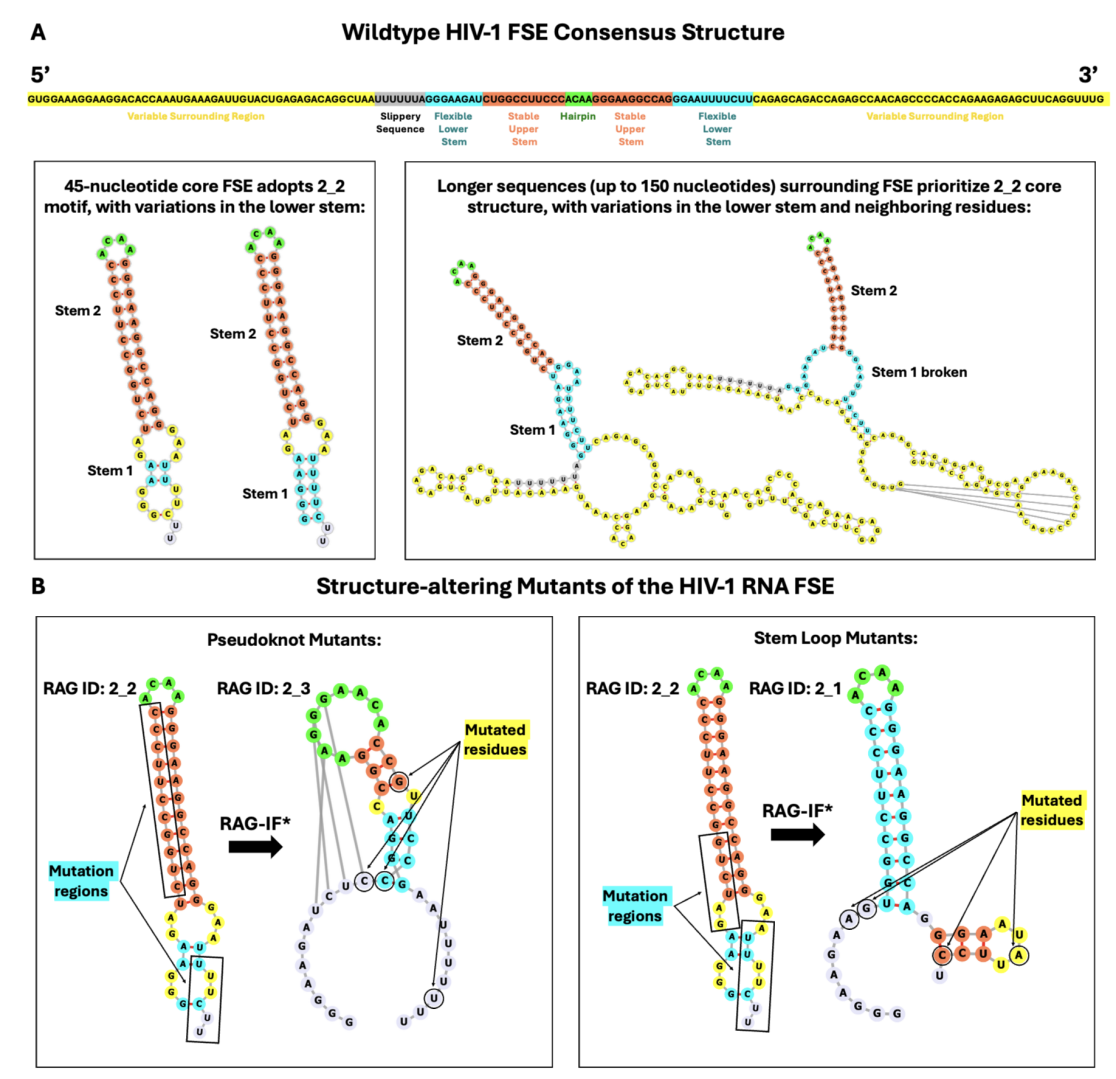
Consensus structure of the HIV-1 FSE and predicted mutants. (**A**) The consensus structure contains a robust upper stem and hairpin loop, flexible lower stem, and variable third region. The stable upper stem agrees with previous literature, and the associated residues are shown in orange in the consensus structure. We find no evidence of a triplex or pseudoknot associated with this region, which is conserved throughout conformational analysis of neighboring residues. In sequences up to 110 nucleotides, the core FSE adopts a 2_2 dual graph motif, suggesting the presence of a lower stem. This lower stem depends critically on context and different variations of this stem are depicted in blue. The purine bulge suggested by the literature may appear at some lengths, but is not widely supported. The variable conformations of the surrounding residues may adopt a more complex structure, which is explored in our dual graph analysis. (**B**) Our two mutants, designed by identifying vulnerable residues in the upper stem and lower loop; see text and Figure 6 later. The RAG-IF algorithm proposes many minimal mutations; the asterisk denotes that the lowest energy mutant with the fewest number of mutations is depicted to the right of the arrow. Similar to Figure 1, the hairpin loop is green, Stem 1 is blue, Stem 2 is coral, internal loops and bulges are yellow, and additional free nucleotides are lavender in the 45 nucleotide 2D structures. For the 150-nucleotide sequences in panel A, the color scheme follows that of the nucleotide sequence found above the boxes (the primary structure of the HIV-1 FSE).

We launch, here, such a frameshifting study on the HIV-1 FSE, a 45-nucleotide sequence [41], without the seven-residue slippery sequence immediately upstream; see Figure 1. Experiments and modeling have suggested different folds for this core region with and without the slippery sequence, including a hairpin loop, stem loop, bulge, and three-way junction, as summarized in Table 1. Whole genome data are also available. We use 2D structure prediction programs with and without restraints from chemical mapping data to examine the HIV-1 FSE folds at lengths varying from 45 to 150 nucleotides to understand the effect of neighboring residues on FSE fold. In particular, we assess our structures at 45 and 150 nucleotides with respect to experimental data [20,34]. This analysis leads us to suggest a consensus fold as shown in Figure 2, with a highly stable upper stem and flexible lower stem as well as a connective loop and three-way junction that anchor other folds (including a pseudoknot and more stem loops) at increasing lengths. We find no evidence for pseudoknot/triplex interactions or stable bulges in the core region. By identifying highly fragile residues in the upper stem and loop regions, we also design minimal mutations using our inverse-folding procedure, RAG-IF. The two mutant products—a pseudoknot and a simple stem loop—alter the natural FSE fold (from stem loop 2_2 to pseudoknot 2_3 or two-stem 2_1 dual graphs, respectively) and define new systems for further experimental tests; see methods on notation—these graph labels V_n refer to RNA graphs of *V* vertices and motif index *n*. Together with the mechanistic insights on HIV-1 frameshift, these findings define subjects for further assessment by all-atom simulations and experimental testing.

**Table 1:** Summary of existing research on the structure of the HIV-1 frameshift element (FSE).

Core FSE Structure	Experimental Method	FSE Length	Key Results	Reference
Simple hairpin loop with *single upper stem*	Mutagenesis and amino acid sequencing	39 nt	Made mutations to slippery sequence, determined slippery sequence is necessary for frameshifting and the stem loop may also be influential	[26]
Stable upper stem with hairpin loop and possible *triplex structure*	Nuclease mapping and frameshift assays	52 nt	Disruption of the proposed triplex-structure resulted in statistically significant decrease in frameshifting efficiency	[31]
	Optical tweezers	52 nt	Upper stem has heterogeneous refolding dynamics, pseudoknot-like triplex can form with truncated version of entire sequence but appears to be rare	[42]
Two-stem helix with *three-purine bulge*	Mutagenesis and enzymatic probing	52 nt	Mutations to the upper stem impacted frameshifting efficiency more than lower stem mutations	[32]
	NMR	45 nt	GC to AU mutations throughout FSE suggest stem stability crucial for frameshifting efficiency	[41]
Two-stem helix with *three-purine bulge*	NMR	41 nt	NMR structure correlates with chemical probing, reiterates upper stem is highly conserved	[43]
	Cryo-EM, NMR, MD	47 nt	Obtained model of HIV-1 RNA duplex, observed super helical twist and flipped out base	[44]
*Three-helix junction* including core hairpin loop	SHAPE	Full genome	FSE upper stem is highly conserved, three-helix junction suggested in broader context	[34]
	Frameshift assay	52 nt	Mutations to the upper stem suggest stability of the first 3–4 base pairs of stem loop is primary determinant of frameshifting efficiency	[45]
	SHAPE	140–160 nt	Identified dynamic switching between different conformations, suggested the global sequence context influences RNA structure	[20]
	NMR	41 nt	RNA switches from three-helix junction to two-helix junction containing an upper and lower stem separated by a purine bulge, average inter-helical bend of 44°	[46]

## 2. Results

### 2.1. SHAPE-Free vs. SHAPE-Guided Characterization of Wildtype HIV-1 FSE Structure

The shape of the HIV-1 frameshift element (FSE) and its surrounding residues has been characterized by several groups and techniques (Table 1). In general, the core FSE (45–52 nt) is found to fold onto a stem loop structure.

The simplest model of the core FSE contains the seven-nucleotide slippery sequence, followed by a spacer region and a single hairpin (Stem 2 in Figure 1) [26,45]. In NMR structures [32,33], the spacer region base pairs with downstream residues, forming an additional stem (Stem 1 in Figure 1). SHAPE data suggest that the slippery sequence base pairs with upstream residues and the spacer region is free. However, an alternate lower stem and an additional anchoring helix are introduced [20]. This three-helix junction was suggested to be unlikely in the natural environment where magnesium ions anchor the bulge that separates the two stems. A two-helix junction was then presented as the most relevant fold [46].

By performing SHAPE-free predictions and SHAPE-guided predictions for short and long, 45 and 150 nucleotide, sequences, along with our conformational landscape from 52 to 150 nucleotides, we can synthesize all existing data (Table 1) and suggest a consensus model (Figure 2). We used 45 nucleotides for the short sequence, as this length is the smallest core FSE that folds onto a stable 2D conformation. Fifty-two nucleotides are often also included in short sequence representations of the FSE (See Table 1), but here, we omit the slippery sequence to focus on the downstream stimulatory structure. The 150-nucleotide length is motivated by the SHAPE study for 140–160 nucleotides (Table 1). Low et al. [20] report a 140-nucleotide region surrounding the FSE, but several of their figures contain up to 160 nucleotide sequences that include a lower anchoring helix. We, thus, chose 150 nucleotides to ensure that any base-pairing residues near the anchoring helix are not cut off.

The structure of the 45-nucleotide sequence is shown in Figure 1B for SHAPE-free prediction and Figure 1C for SHAPE-guided prediction. Both structures fold onto the 2_2 stem-loop dual graph motif and yield a four-nucleotide hairpin (ACAA) with a highly conserved upper stem (Stem 2). The literature repeatedly reports the presence of this hairpin and upper stem [20,32,45]. Conversely, Stem 1 is a small, flexible structure that is separated from Stem 2 by an asymmetric internal loop in both SHAPE-free and SHAPE-guided prediction. Stem 1 is much shorter in the SHAPE-free model, and the flexibility and instability of this lower stem is explored in the conformational landscape. Previous studies have reported a three-purine bulge in place of the internal loop (See Table 1), but our findings do not support such a structure within the 45-nucleotide core FSE.

**Figure 3:**
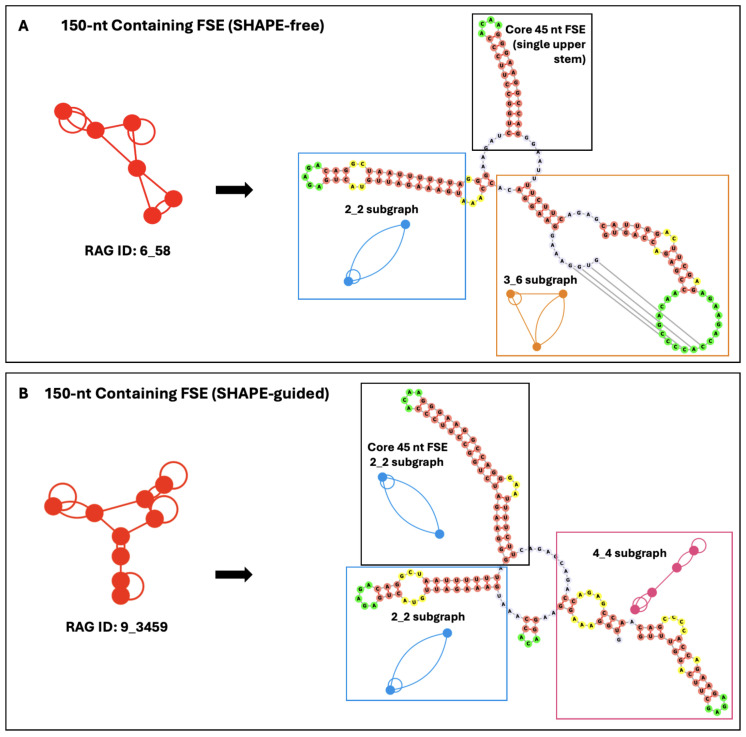
SHAPE-free (**A**) and SHAPE-guided (**B**) secondary structure prediction of the HIV-1 FSE when expanded to 150 nucleotides. The SHAPE-free data predict a 6_58 dual graph (composed of a 2_2 stem loop, 2_1 stem, and 3_6 pseudoknot), while the SHAPE-guided data predict a 9_3459 dual graph (composed of two 2_2 stem loops and a four-stem loop structure). Key sub-graphs for each are highlighted, with the accompanying dual graph motifs shown. All hairpin turns are green, base-pairing regions are coral, internal loops and bulges are yellow, and additional free nucleotides are lavender. See notation in Methods and Figure 4 for sub graphs mentioned.

Figure 3 presents folds of the FSE in a longer construct of 150 nucleotides without (top) and with (bottom) SHAPE-guided experimental restraints from [34]. The ACAA hairpin and surrounding stem are maintained as in the 45-nucleotide system. The greatest variation in the literature comes with how the FSE folds within the surrounding nucleotides. In the SHAPE-free model, the lower stem of the 2_2 dual graph disappears, and the core FSE contains a single upper stem. The slippery sequence engages with upstream residues to form a 2_2 stem-loop. A three-helix junction connects these two structures (the stable hairpin of the core FSE and the 2_2 motif containing the slippery sequence) to a 3_6 pseudoknot. Interestingly this is the same pseudoknot motif found in the SARS-CoV-2 FSE [39]. However, with SHAPE-guided experimental restraints, the core FSE folds onto a 2_2 dual graph, as in the 45-nucleotide predictions. The lower stem is slightly longer than either short sequence predictions and a three-purine bulge emerges, consistent with some of the previously mentioned literature [32]. The slippery sequence folds onto a 2_2 stem-loop with upstream residues, similar to the SHAPE-free prediction. The 3_6 pseudoknot predicted above between residues upstream and downstream from the core FSE is absent; instead, simple stem-loops are formed from the surrounding elements.

Both SHAPE-free and SHAPE-guided predictions at 45 and 150 nucleotides are synthesized in Figure 2. The 2_2 motif of the core FSE appears to be robust and insensitive to increases in neighboring residues. The lower stem of the core FSE is flexible, and the variation between prediction methods indicates lower stability of this lower stem, as compared to the crucial upper stem and hairpin loop. Conformational analysis elaborates on these findings.

### 2.2. Conformational Analysis of HIV-1 FSE and Nearby Residues

**Figure 4:**
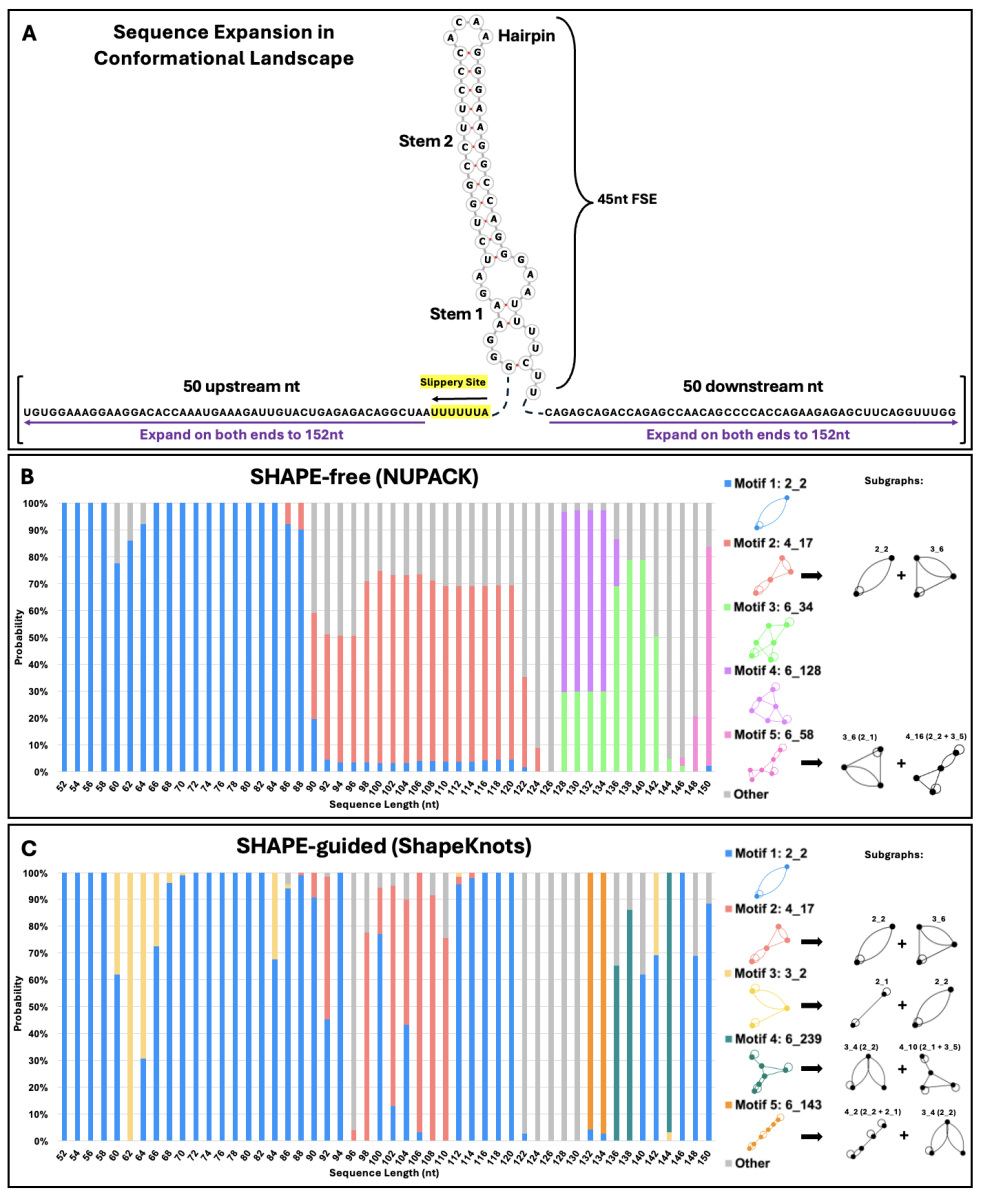
HIV-1 conformational landscape analysis. (**A**) Bidirectional conformational landscape of the HIV-1 RNA FSE. First, the slippery sequence is added one residue at a time (highlighted in yellow), for a total of seven nucleotides. Then, nucleotides are added two at a time, one upstream and one downstream of the FSE, until the sequence is 152 nucleotides in length. The top five most common dual graph motifs in conformational analysis are presented in (**B**,**C**), along with major sub-graphs of larger motifs. These subgraphs are obtained by our partitioning program, which keeps junctions and pseudoknots intact [48]. The probability of each possible fold at each length is plotted on the vertical axis, with the length of the sequence along the horizontal axis. (**B**) depicts the conformational analysis using NUPACK and (**C**) Conformational analysis using ShapeKnots.

Conformational analysis can provide insight into the fold of the frameshifting region as the neighboring residues change, highlighting key structural elements and dynamics of the FSE [39]. Figure 4A depicts how sequence is increased: Each residue of the slippery sequence is added and, then, two residues are added at a time, one upstream and one downstream (symmetric expansion). In Figure 4B,C, we show the results for SHAPE-free versus SHAPE-guided predictions as length changes. At each length, the color bars denote the percentage of each motif present, and the motifs, and their subgraphs, are shown at right. The presence of blue lines highlights the robustness of the 2_2 stem-loop motif. Without SHAPE, 2_2 is stable until approximately 90 nucleotides, and with SHAPE, a pseudoknot fold emerges at limited lengths then disappears. Beyond 90 nucleotides, the lower stem of the 2_2 subgraph grows into 6_34 and 6_128 motifs, which dominate lengths of 128 to 142 nucleotides, and a 6_58 dual graph (pseudoknot containing motif) at 150 nucleotides, consistent with Figure 3. With SHAPE-guided data, the 2_2 stem-loop is more stable, with fewer pseudoknot-containing structures. In the 3_2, 6_239 and 6_143 motifs (Figure 4), the upper stem and hairpin loop of the 2_2 motif are maintained, despite variations in the lower stem. The core FSE adopts a 2_2 sub-graph within four of the top five motifs using SHAPE-guided data.

**Figure 5:**
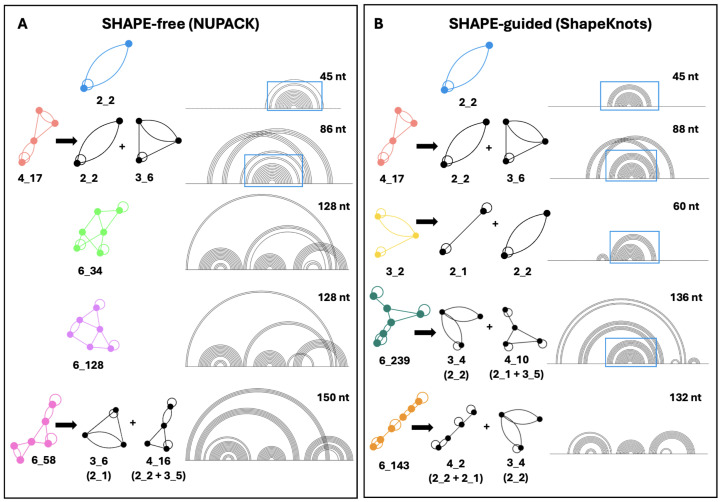
The top five dual graph motifs in the SHAPE-free (**A**) and SHAPE-guided (**B**) conformational landscapes, accompanied by major sub-graphs [48]. The associated dual graph is depicted on the left, with the arc plot to the right. The top five motifs from most to least common for SHAPE-free prediction (**A**) are 2_2, 4_17, 6_34, 6_128, and 6_58. The top five motifs from SHAPE-guided data (**B**) are 2_2, 4_17, 3_2, 6_239, and 6_143. When the 45-nucleotide FSE folds onto a 2_2 dual graph, the associated base pairs are boxed in blue. Associated subgraphs are shown by graph partitioning [48].

Besides the robustness of the 2_2 stem-loop, the second most common motif in both landscapes is the pseudoknot-containing 4_17 dual graph. This motif is composed of the 3_6 pseudoknot found in the SARS-CoV-2 FSE [39], plus the 2_2 stem loop. The ACAA hairpin and upper stem in Figure 1 are maintained in these pseudoknot containing structures. The persistence of this crucial hairpin and base-pairing region can be observed in Figure 5, which elaborates on the folds obtained in the conformational landscape. Despite fluidity in the secondary structure surrounding these residues, a nearly identical stem and hairpin appear in all of the top five motifs in both conformational landscapes. In SHAPE-free predictions of longer sequences, several pseudoknots are introduced, but they are essentially modular and independent of the 45 nucleotides in the FSE, as some studies have suggested [32]. The SHAPE-free landscape shows the presence of an upstream 2_2 subgraph in motifs 6_34, 6_128, and 6_58 (Figure 5). These structures may play a role in translation, interacting with the ribosome before it reaches the FSE, as the slippery sequence is based paired to upstream residues in the lower stem of this structural element. These motifs appear as the fifth most common motif, 6_143, in the SHAPE-guided landscape, energetically favorable at sequence lengths of 132 and 134 nucleotides.

### 2.3. Mutations for Target Graph 2_3 to Introduce Pseudoknot

The rationale for mutation design is to identify minimal mutants that fold onto other target motifs than the wildtype 2_2 as candidates for further all-atom simulations and experimental analysis, following our successful strategy for SARS-CoV-2 [28,39]. For the SARS-CoV-2 FSE, the three RAG-IF-designed mutants that stabilized 3_6, 3_3, and 3_5 motifs suppressed conformational transitions and nearly abolished frameshifting [28].

**Figure 6:**
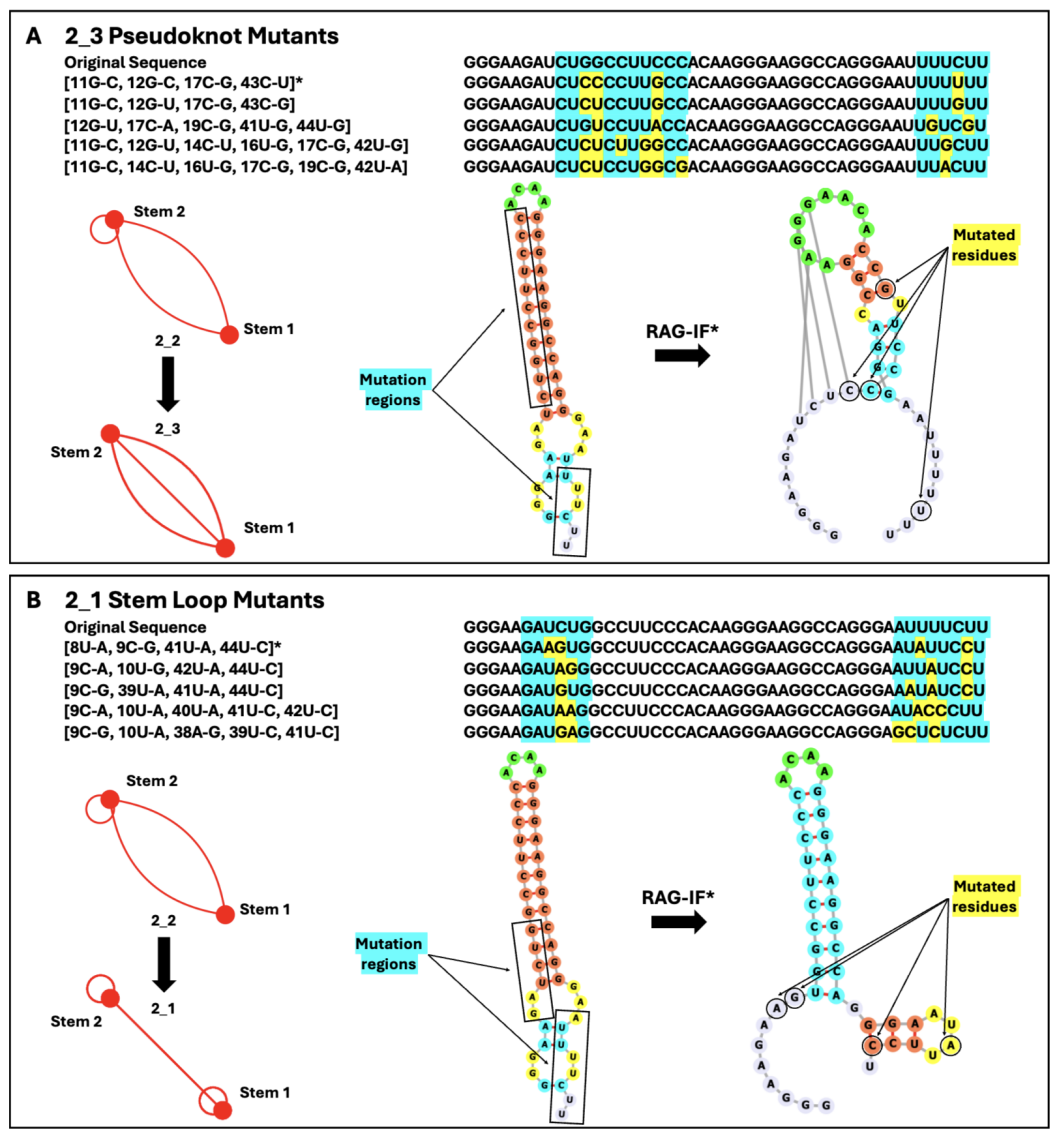
Mutation results for target graphs 2_3 (**A**) and 2_1 (**B**). The original sequence is listed at the top of each panel followed by the top five minimal mutations that enact the desired changed in dual graph topology. The target mutation region for RAG-IF is highlighted in blue and the mutated residues are highlighted in yellow. The asterisk denotes the lowest energy mutant with the fewest number of mutations; this mutant is depicted below the sequences in each panel. The original and mutant dual graph and secondary structure are depicted for each. Here, the mutation region is boxed and the mutated residues are circled. Folding was predicted by NUPACK and IPknots. The ACAA hairpin loop is in green, Stem 1 is blue, Stem 2 is coral, additional internal loops are yellow, and additional free nucleotides are lavender in 2D structures.

The first mutant here was designed to alter the secondary structure of the HIV-1 RNA FSE from a 2_2 dual graph to the pseudoknot-containing 2_3 dual graph (Figure 6). The original secondary structure of the FSE was a simple two-stem structure containing a highly conserved hairpin and upper stem. We hypothesize that the introduction of a bulky pseudoknot could interrupt normal frameshifting patterns. In our target transformation from 2_2 to 2_3, we break the upstream portion of the lower stem to allow the downstream residues of the preexisting stem to form a pseudoknot with residues that are a part of the upper hairpin. The minimal number of mutations required to produce this pseudoknot instead of a stem-loop is four: [11G-C, 12G-C, 17C-G, 43C-U]. A similar transformation can be achieved mutating 12G-U and 43C-G, but the former mutant is more stable. That is because the highly conserved ACAA hairpin loop is expanded and forms a pseudoknot with downstream residues towards the end of the FSE.

### 2.4. Mutations for Target Graph 2_1 to Disrupt Lower Stem

The second mutant is intended to change the secondary structure of the FSE from a 2_2 stem-loop to a simple two-stem topology (2_1 dual graph). This transformation is achieved with four minimal mutations [8U-A, 9C-G, 41U-A, 44U-C]. This mutant disrupts the lower stem in the wildtype HIV-1. The highly conserved ACAA hairpin remains intact, but we introduce a second hairpin further downstream. The transformation to a 2_1 dual graph can also be accomplished by two other minimal mutations of four residues: [9C-A, 10U-G, 42U-A, 44C-C], and [9C-G, 39U-A, 41U-A, 44U-C]. While IPknots predicted some of these mutant sequences to fold into the 3_6 psuedoknot instead, the 2_1 dual graph is more stable because an additional four-nucleotide hairpin is introduced (Figure 6).

## 3. Discussion

We have analyzed the structure of the HIV-1 frameshift element (FSE) and proposed structure-altering mutations using computational tools and our RAG database. Our conformational analysis shows that, despite the persistence of the hairpin loop (2_2 dual graph stem-loop), the HIV-1 FSE is capable of adapting other forms, especially when surrounded by more residues. Given experiments suggesting that the FSE fold affects the efficiency of the -1 PRF mechanism [35,43], also for SARS-CoV-2 [10,27,28], altering FSE folds using mutations or small drugs define new therapeutic avenues.

Our landscapes suggest folds for the core FSE (Figure 2), where the dominant structure is a simple hairpin loop, or 2_2 dual graph. The lower stem in this stem-loop structure is fragile, while the upper stem and hairpin loop appear robust, recurring in nearly all lengths of our conformational analysis. This finding is well supported by the literature [19,20,43]. We do not, however, find evidence of stable bulges, triplexes, or pseudoknots in the core FSE, as some studies have suggested [31], and this should be further evaluated experimentally.

Our conformational landscapes highlight the disruption and regeneration of key structural motifs in the residues surrounding the HIV-1 FSE during ribosomal frameshifting. Key structural elements, as presented in Figure 2, define potential targets for mutations or small drug binding sites. Our two mutants (Figure 6) define subjects for further simulation and experiment. Our first mutant introduces a pseudoknot to the highly conserved hairpin loop of the frameshift stimulatory sequence, changing the secondary structure from a 2_2 to 2_3 dual graph motif. From our conformational analysis, this crucial hairpin contains four nucleotides in nearly all lengths. The 2_3 mutant has a six-nucleotide hairpin loop, and a symmetrical, one-nucleotide bulge in the original upper stem of the wildtype structure (Figure 1). The local stability of the first three to four base pairs in the upper stem has been indicative of frameshift efficiency [45], particularly, strong G–C pairs [49]. It is believed that the ribosome interacts with the readily breakable lower stem, while this upper stem remains intact for much of translation [50]. The disruption of the fourth base pair of the original upper stem, in addition to the insertion of a pseudoknot, may influence stability of the FSE and the frameshift efficiency [51].

Our second mutant (Figure 6) maintains the stable upper stem in the wildtype HIV-1 FSE, frees up the spacer region, and introduces a novel downstream stem. This 2_1 motif was found to be a minor contributor to the overall patterns in secondary structure evaluated in conformational analysis. The lower stem that is broken in the 2_1 mutant has been hypothesized to be important to frameshifting because it would be the first to be unwound during translation [52] and is known to be unfolded by ribosomes with ease [42,50]. This is interesting considering that the first three base pairs are G–C pairs. It is possible that removal of the unstable lower stem would hasten the ribosome’s interaction with the spacer region and alter the stability of the upper stem. The deletion of a nucleotide in the spacer region has been associated with a decrease in the stability of the downstream stem loop [45], which is a known factor of frameshift efficiency.

It is intriguing that while HIV-1 ribosomal frameshifting process appears to be stimulated by a simple hairpin, as opposed to a more complex three-junction structure or pseudoknot, its FSE still has complex folding dynamics, as indicated by the literature [42] and the flexibility seen in our conformational landscape. Related all-atom molecular dynamics and enhanced sampling simulations of the HIV-1 FSE wildtype system and mutants, along with chemical reactivity and functional characterization experiments, are needed to further explore these findings, including our mutants, as potential therapeutic targets.

## 4. Materials and Methods

### 4.1. Identification of the HIV-1 RNA FSE

The site of Gag/Gag-Pol frameshifting has been well defined. The HIV-1 RNA frameshift element (FSE) has the slippery sequence UUUUUUA that precedes the downstream stimulatory sequence. The FSE is defined as the 45 nucleotides that make up this stimulatory region from residues 1638 to 1683 from SHAPE data of the entire HIV-1 genome [20]. The location of the FSE with respect to the HIV-1 genome is depicted in Figure 1.

### 4.2. Secondary Structure Prediction Packages

Three different secondary structure prediction packages are used in the present study. For dual graph representation and the creation of mutants, NUPACK [53] and IPknot [54] were utilized. The RAG-IF code can also use PKNOTs [55] if specified. For conformational analysis, ShapeKnots [56] was used when SHAPE data for the HIV-1 genome were available as restraints to the 2D structure prediction. These choices are based on our prior experience with SARS-CoV-2 and the extensive comparative analysis for different programs [10,28,29] as well as computational performance, ability to predict pseudoknots, and ability to incorporate chemical mapping data as restraints for 2D folding.

### 4.3. RAG Dual Graphs

Our dual graph representation of RNA guided by graph theory [37,38,57,58] is used to describe folding motifs in a way not sensitive to small changes in stem or loop length or content; all 2D folding is still performed in full nucleotide space. The RAG dual graph motifs are identified by the number of stems present in the defined region of the RNA, represented by vertices, and connectivity of the stems is represented by edges. In dual graphs, a vertex represents a stem with at least two base pairs. Edges denote strands and loops. Hairpins are depicted as self loops, and one-nucleotide bulges or internal loops with one-nucleotide strands are ignored. Dual graphs are labeled as V_n where *V* represents the number of vertices (or stems) and *n* is an index unique to each motif [38] (See Figure 1 and Figure 3 for examples). The RAG dual graph library enumerates over 100,000 motifs [57,58]. By using a motif-based notation rather than nucleotide focused notation, we can group, under the same motif ID, different folds that share topological features, despite differences in size and content of individual elements. Subgraphs can also be obtained for larger dual graphs [48]. For full details, see the cited references.

### 4.4. Conformational Landscapes and SHAPE Data

We investigate how sequence context may influence folding patterns in the FSE by creating a conformational landscape, as introduced by Yan and Schlick [29,38,39], by embedding the FSE in larger sequence frames. The bidirectional conformational analysis is motivated by the desire to understand how the RNA neighboring sequences affect the folding. If we were mimicking ribosome translation, residues should be added only on the 5′ end (asymmetric upstream) [19,27,38]. Conversely, landscapes with added 3′ (asymmetric downstream) residues would mimic refolding when ribosomes move further downstream. We have reported such unidirectional landscapes in our prior works; see, for example [27].

Conformational landscapes were generated in two ways: by pure computational (SHAPE-free) prediction using NUPACK [53], and with SHAPE-guided 2D structure prediction using ShapeKnots [56]. The SHAPE data are taken from whole genome mapping data [20,34]. When increasing the sequence length, we add one residue at a time for the slippery sequence and, then, two at a time (one upstream and one downstream) until the desired sequence length was reached. The bidirectional analysis presented here is useful in understanding the general effect of the FSE surroundings on the fold [29]. We note, however, that during translation, multiple ribosomes may occlude different parts of the mRNA that are in the ribosomal channel or other regions near a ribosome. Thus, some secondary structures that emerge in free RNA will not be possible. These scenarios are difficult to account for by both modeling and structure determination. Thus, some folds presented here may not be possible in vivo.

### 4.5. RAG-IF for Minimal Mutations

The RAG-IF algorithm [10,57] has three key steps: identify a mutation region and target dual graph, produce mutations in the defined region using a genetic algorithm, and organize the pool of mutants according to the minimum number of mutations required to achieve the target structure. This computational method uses a genetic algorithm to introduce mutations into the RNA sequence and find the minimum number of mutations necessary to achieve a desired change in the motif describing the secondary structure of the viral RNA [57]. The RAG-IF code creates hundreds of candidate sequences that are organized by minimal mutations. Dramatic structural changes can be achieved with relatively small amounts of point mutations [10]. The mutation region can be defined by the user or performed automatically. We use automatic design for the 45-residue sequence for HIV-1. The RAG-IF algorithm ran for less than three hours for both of the mutants presented in this paper.

Initially, we focus on breaking the lower stem, thus creating a simple stem-loop structure (2_1) and intertwining the two stems to create a pseudoknot (2_3). The genetic algorithm imitates evolution in nature to introduce point mutations in the RNA sequence. After random mutations, crossover events, and selection, sequences that match the target structure are kept. NUPACK is used for initial screening of secondary structure [53]. Surviving sequences are checked again using IPknot [54]. Only sequences that achieve the target dual graph structure with both prediction programs are retained and are sorted by the minimum number of mutations required to induce the desired change in secondary structure, per our RAG database [57,58]. As mentioned above, the three mutants we created for the SARS-CoV-2 FSE were experimentally verified to fold onto target motifs stably, that is, without alternative folds, and resulted in lower frameshifting by an order of magnitude [28].

## Data Availability

No new data were created or analyzed in this study. Data sharing is not applicable to this article.

## References

[1] van Schalkwyk C., Mahy M., Johnson L.F., Imai-Eaton J.W. (2024). Updated data and methods for the 2023 UNAIDS HIV estimates. JAIDS.

[2] Gumna J., Antczak M., Adamiak R.W., Bujnicki J.M., Chen S.J., Ding F., Ghosh P., Li J., Mukherjee S., Nithin C. (2022). Computational Pipeline for Reference-Free Comparative Analysis of RNA 3D Structures Applied to SARS-CoV-2 UTR Models. Int. J. Mol. Sci..

[3] Li Q., Wang Q., Wang R., Zhang L., Liu Z. (2025). The frameshifting element in coronaviruses: Structure, function, and potential as a therapeutic target. Trends Sci..

[4] Dinman J.D. (2006). Programmed Ribosomal Frameshifting Goes Beyond Viruses: Organisms from all three kingdoms use frameshifting to regulate gene expression, perhaps signaling a paradigm shift. Microbe.

[5] Caliskan N., Peske F., Rodnina M.V. (2015). Changed in translation: mRNA recoding by -1 programmed ribosomal frameshifting. Trends Biochem. Sci..

[6] Atkins J.F., Loughran G., Bhatt P.R., Firth A.E., Baranov P.V. (2016). Ribosomal frameshifting and transcriptional slippage: From genetic steganography and cryptography to adventitious use. Nucleic Acids Res..

[7] Kobayashi Y., Zhuang J., Peltz S., Dougherty J. (2010). Identification of a cellular factor that modulates HIV-1 programmed ribosomal frameshifting. J. Biol. Chem..

[8] Anokhina V.S., Miller B.L. (2021). Targeting Ribosomal Frameshifting as an Antiviral Strategy: From HIV-1 to SARS-CoV-2. Acc. Chem. Res..

[9] Wang X., Xuan Y., Han Y., Ding X., Ye K., Yang F., Gao P., Goff S.P., Gao G. (2019). Regulation of HIV-1 Gag-Pol Expression by Shiftless, an Inhibitor of Programmed -1 Ribosomal Frameshifting. Cell.

[10] Schlick T., Zhu Q., Jain S., Yan S. (2021). Structure-altering mutations of the SARS-CoV-2 frameshifting RNA element. Biophys. J..

[11] Bhatt P.R., Scaiola A., Loughran G., Leibundgut M., Kratzel A., Meurs R., Dreos R., O’Connor K.M., McMillan A., Bode J.W. (2021). Structural basis of ribosomal frameshifting during translation of the SARS-CoV-2 RNA genome. Science.

[12] Zhang K., Zheludev I.N., Hagey R.J., Haslecker R., Hou Y.J., Kretsch R., Pintilie G.D., Rangan R., Kladwang W., Li S. (2021). Cryo-EM and antisense targeting of the 28-kDa frameshift stimulation element from the SARS-CoV-2 RNA genome. Nat. Struct. Mol. Biol..

[13] Kelly J.A., Olson A.N., Neupane K., Munshi S., San Emeterio J., Pollack L., Woodside M.T., Dinman J.D. (2020). Structural and functional conservation of the programmed -1 ribosomal frameshift signal of SARS coronavirus 2 (SARS-CoV-2). J. Biol. Chem..

[14] Lan T.C.T., Allan M.F., Malsick L.E., Woo J.Z., Zhu C., Zhang F., Khandwala S., Nyeo S.S.Y., Sun Y., Guo J.U. (2022). Secondary structural ensembles of the SARS-CoV-2 RNA genome in infected cells. Nat. Commun..

[15] Huston N.C., Wan H., Strine M.S., de Cesaris Araujo Tavares R., Wilen C.B., Pyle A.M. (2021). Comprehensive in vivo secondary structure of the SARS-CoV-2 genome reveals novel regulatory motifs and mechanisms. Mol. Cell.

[16] Jones C.P., Ferré-D’Amaré A.R. (2021). Crystal structure of the severe acute respiratory syndrome coronavirus 2 (SARS-CoV-2) frameshifting pseudoknot. RNA.

[17] Weichseldorfer M., Reitz M., Latinovic O.S. (2021). Past HIV-1 medications and the current status of combined antiretroviral therapy options for HIV-1 patients. Pharmaceutics.

[18] Deeks S.G. (2003). Treatment of antiretroviral-drug-resistant HIV-1 infection. Lancet.

[19] Brierley I., Dos Ramos F.J. (2006). Programmed ribosomal frameshifting in HIV-1 and the SARS–CoV. Virus Res..

[20] Low J.T., Garcia-Miranda P., Mouzakis K.D., Gorelick R.J., Butcher S.E., Weeks K.M. (2014). Structure and dynamics of the HIV-1 frameshift element RNA. Biochemistry.

[21] Shehu-Xhilaga M., Crowe S.M., Mak J. (2001). Maintenance of the Gag/Gag-Pol ratio is important for human immunodeficiency virus type 1 RNA dimerization and viral infectivity. J. Virol..

[22] Hilimire T.A., Chamberlain J.M., Anokhina V., Bennett R.P., Swart O., Myers J.R., Ashton J.M., Stewart R.A., Featherston A.L., Gates K. (2017). HIV-1 frameshift RNA-targeted triazoles inhibit propagation of replication-competent and multi-drug-resistant HIV in human cells. ACS Chem. Biol..

[23] German Advisory Committee Blood (Arbeitskreis Blut), Subgroup ‘Assessment of Pathogens Transmissible by Blood’ (2016). Human immunodeficiency virus (HIV). Trans. Med. Hemother..

[24] Biswas P., Jiang X., Pacchia A.L., Dougherty J.P., Peltz S.W. (2004). The human immunodeficiency virus type 1 ribosomal frameshifting site is an invariant sequence determinant and an important target for antiviral therapy. J. Virol..

[25] Wilson W., Braddock M., Adams S.E., Rathjen P.D., Kingsman S.M., Kingsman A.J. (1988). HIV expression strategies: Ribosomal frameshifting is directed by a short sequence in both mammalian and yeast systems. Cell.

[26] Jacks T., Madhani H.D., Masiarz F.R., Varmus H.E. (1988). Signals for ribosomal frameshifting in the Rous sarcoma virus gag-pol region. Cell.

[27] Lee S., Yan S., Dey A., Laederach A., Schlick T. (2025). A Cascade of Conformational Switches in SARS-CoV-2 Frameshifting: Coregulation by Upstream and Downstream Elements. Biochemistry.

[28] Dey A., Yan S., Schlick T., Laederach A. (2024). Abolished frameshifting for predicted structure-stabilizing SARS-CoV-2 mutants: Implications to alternative conformations and their statistical structural analyses. RNA.

[29] Schlick T., Zhu Q., Dey A., Jain S., Yan S., Laederach A. (2021). To knot or not to knot: Multiple conformations of the SARS-CoV-2 frameshifting RNA element. J. Amer. Chem. Soc..

[30] Le S.Y., Shapiro B.A., Chen J.H., Nussinov R., Maizel J.V. (1991). RNA pseudoknots downstream of the frameshift sites of retroviruses. Genet. Anal. Biomol. Eng..

[31] Dinman J.D., Richter S., Plant E.P., Taylor R.C., Hammell A.B., Rana T.M. (2002). The frameshift signal of HIV-1 involves a potential intramolecular triplex RNA structure. Proc. Natl. Acad. Sci. USA.

[32] Dulude D., Baril M., Brakier-Gingras L. (2002). Characterization of the frameshift stimulatory signal controlling a programmed −1 ribosomal frameshift in the human immunodeficiency virus type 1. Nucleic Acids Res..

[33] Baril M., Dulude D., Gendron K., Lemay G., Brakier-Gingras L. (2003). Efficiency of a programmed −1 ribosomal frameshift in the different subtypes of the human immunodeficiency virus type 1 group M. RNA.

[34] Watts J.M., Dang K.K., Gorelick R.J., Leonard C.W., Bess J.W., Swanstrom R., Burch C.L., Weeks K.M. (2009). Architecture and secondary structure of an entire HIV-1 RNA genome. Nature.

[35] Garcia-Miranda P., Becker J.T., Benner B.E., Blume A., Sherer N.M., Butcher S.E. (2016). Stability of HIV frameshift site RNA correlates with frameshift efficiency and decreased virus infectivity. J. Virol..

[36] Gan H.H., Pasquali S., Schlick T. (2003). Exploring the repertoire of RNA secondary motifs using graph theory; implications for RNA design. Nucleic Acids Res..

[37] Meng G., Tariq M., Jain S., Elmetwaly S., Schlick T. (2020). RAG-Web: RNA structure prediction/design using RNA-As-Graphs. Bioinformatics.

[38] Yan S., Zhu Q., Jain S., Schlick T. (2022). Length-dependent motions of SARS-CoV-2 frameshifting RNA pseudoknot and alternative conformations suggest avenues for frameshifting suppression. Nat. Commun..

[39] Yan S., Schlick T. (2025). Heterogeneous and multiple conformational transition pathways between pseudoknots of the SARS-CoV-2 frameshift element. Proc. Natl. Acad. Sci. USA.

[40] Yan S., Zhu Q., Hohl J., Dong A., Schlick T. (2023). Evolution of coronavirus frameshifting elements: Competing stem networks explain conservation and variability. Proc. Natl. Acad. Sci. USA.

[41] Staple D.W., Butcher S.E. (2005). Solution structure and thermodynamic investigation of the HIV-1 frameshift inducing element. J. Mol. Biol..

[42] Ritchie D.B., Cappellano T.R., Tittle C., Rezajooei N., Rouleau L., Sikkema W.K., Woodside M.T. (2017). Conformational dynamics of the frameshift stimulatory structure in HIV-1. RNA.

[43] Gaudin C., Mazauric M.H., Traïkia M., Guittet E., Yoshizawa S., Fourmy D. (2005). Structure of the RNA signal essential for translational frameshifting in HIV-1. J. Mol. Biol..

[44] Zhang K., Keane S.C., Su Z., Irobalieva R.N., Chen M., Van V., Sciandra C.A., Marchant J., Heng X., Schmid M.F. (2018). Structure of the 30 kDa HIV-1 RNA dimerization signal by a hybrid cryo-EM, NMR, and molecular dynamics approach. Structure.

[45] Mouzakis K.D., Lang A.L., Vander Meulen K.A., Easterday P.D., Butcher S.E. (2013). HIV-1 frameshift efficiency is primarily determined by the stability of base pairs positioned at the mRNA entrance channel of the ribosome. Nucleic Acids Res..

[46] Mouzakis K.D., Dethoff E.A., Tonelli M., Al-Hashimi H., Butcher S.E. (2015). Dynamic motions of the HIV-1 frameshift site RNA. Biophys. J..

[47] Lai D., Proctor J.R., Zhu J.Y.A., Meyer I.M. (2012). R-CHIE: A web server and R package for visualizing RNA secondary structures. Nucleic Acids Res..

[48] Jain S., Bayrak C.S., Petingi L., Schlick T. (2018). Dual graph partitioning highlights a small group of pseudoknot-containing RNA submotifs. Genes.

[49] Telenti A., Martinez R., Munoz M., Bleiber G., Greub G., Sanglard D., Peters S. (2002). Analysis of natural variants of the human immunodeficiency virus type 1 gag-pol frameshift stem-loop structure. J. Virol..

[50] Mazauric M.H., Seol Y., Yoshizawa S., Visscher K., Fourmy D. (2009). Interaction of the HIV-1 frameshift signal with the ribosome. Nucleic Acids Res..

[51] Giedroc D.P., Cornish P.V. (2009). Frameshifting RNA pseudoknots: Structure and mechanism. Virus Res..

[52] Seah Y.L. (2017). Molecular Dynamics Simulation of RNA Ribosomal Frameshift Stimulatory Elements. Ph.D. Thesis.

[53] Dirks R.M., Pierce N.A. (2003). A partition function algorithm for nucleic acid secondary structure including pseudoknots. J. Comput. Chem..

[54] Sato K., Kato Y., Hamada M., Akutsu T., Asai K. (2011). IPknot: Fast and accurate prediction of RNA secondary structures with pseudoknots using integer programming. Bioinformatics.

[55] Rivas E., Eddy S.R. (1999). A dynamic programming algorithm for RNA structure prediction including pseudoknots. J. Mol. Biol..

[56] Hajdin C.E., Bellaousov S., Huggins W., Leonard C.W., Mathews D.H., Weeks K.M. (2013). Accurate SHAPE-directed RNA secondary structure modeling, including pseudoknots. Proc. Natl. Acad. Sci. USA.

[57] Jain S., Tao Y., Schlick T. (2020). Inverse folding with RNA-As-Graphs produces a large pool of candidate sequences with target topologies. J. Struct. Biol..

[58] Jain S., Saju S., Petingi L., Schlick T. (2019). An extended dual graph library and partitioning algorithm applicable to pseudoknotted RNA structures. Methods.

